# Supplementary material for: Atomic force microscopy methodology and AFMech Suite software for nanomechanics on heterogeneous soft materials
Source: Nat Commun. 2018 Sep 4;9:3584. doi: 10.1038/s41467-018-05902-1 (PMC6123404; doi:10.1038/s41467-018-05902-1)
Supplement: Supplementary file 1 — Supplementary Information [file 41467_2018_5902_MOESM1_ESM.pdf]

Atomic force microscopy methodology and AFMech Suite software for nanomechanics on heterogeneous soft materials.

Galluzzi et al.

Supplementary information

Supplementary Figures

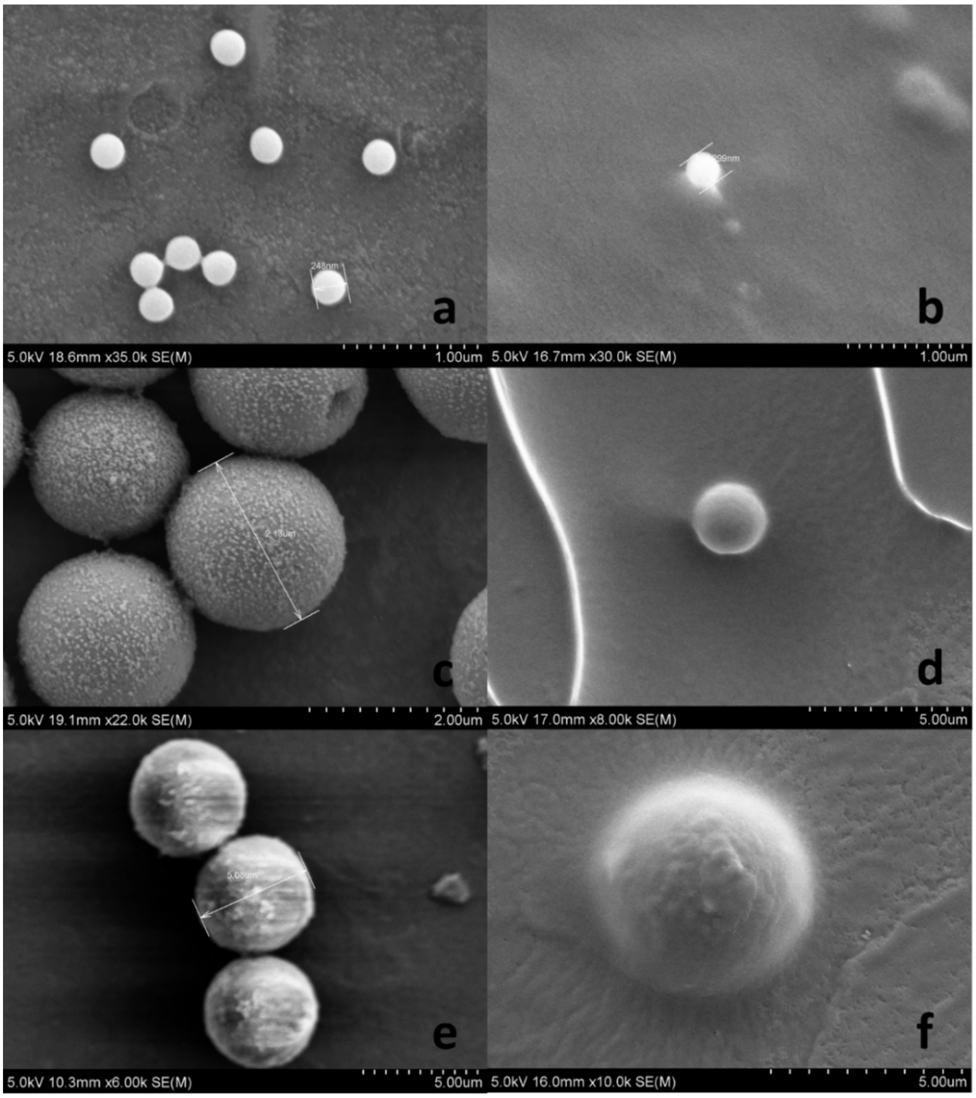

Supplementary Figure 1 | Surface morphology of spheres and composite hydrogels by scanning electron microscopy. Monodispersed polystyrene spheres deposited on glass having different size (**a**) R = 0.15 μm, (**c**) R = 1 μm, (**e**) R = 2.5 μm. In the same order, SEM morphology of freeze-dried composite gels embedded with PS spheres (**b**) R = 0.15 μm, (**d**) R = 1 μm, (**f**) R = 2.5 μm.

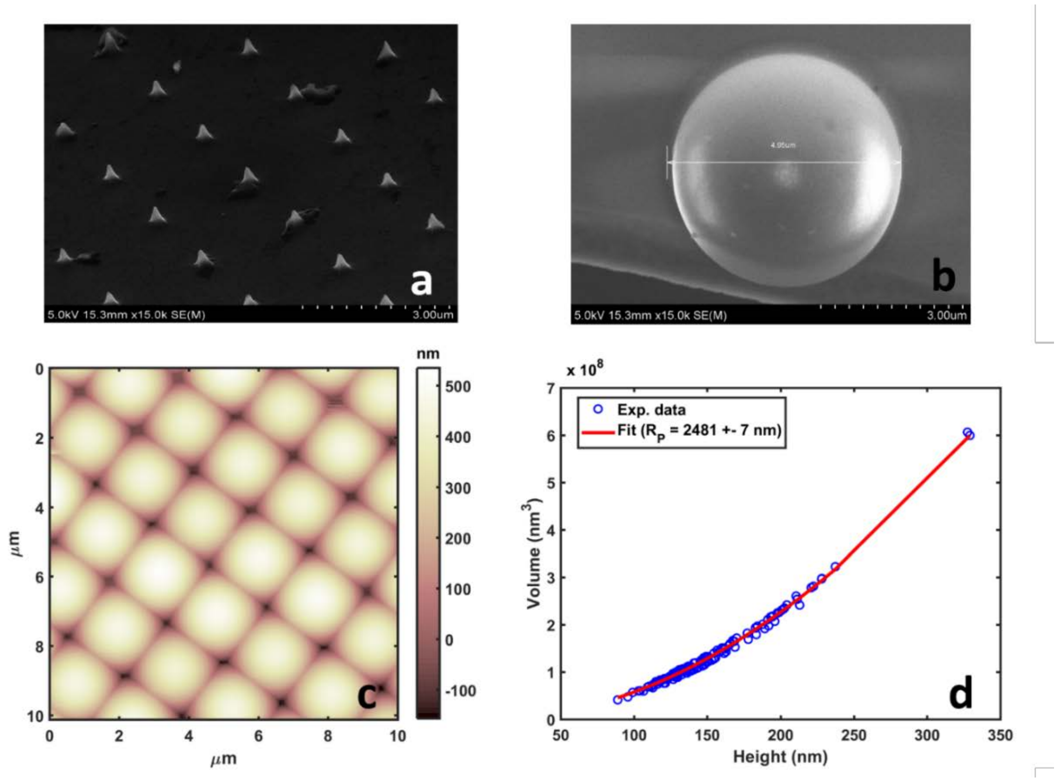

Supplementary Figure 2 | Colloidal probes radius calibration. (**a**) SEM image of surface of calibration sample TGT1 composed by sharp spikes, (**b**) SEM image of spherical colloidal probe (**c**) Reverse AFM imaging morphology of spherical colloidal probe after measurements on the hydrogel series. (**d**) Correlation between volumes and heights of spherical caps leading to probe radius. Experimental errors are comparable with symbol size, while final error of radius is calculated by fitting on experimental data.

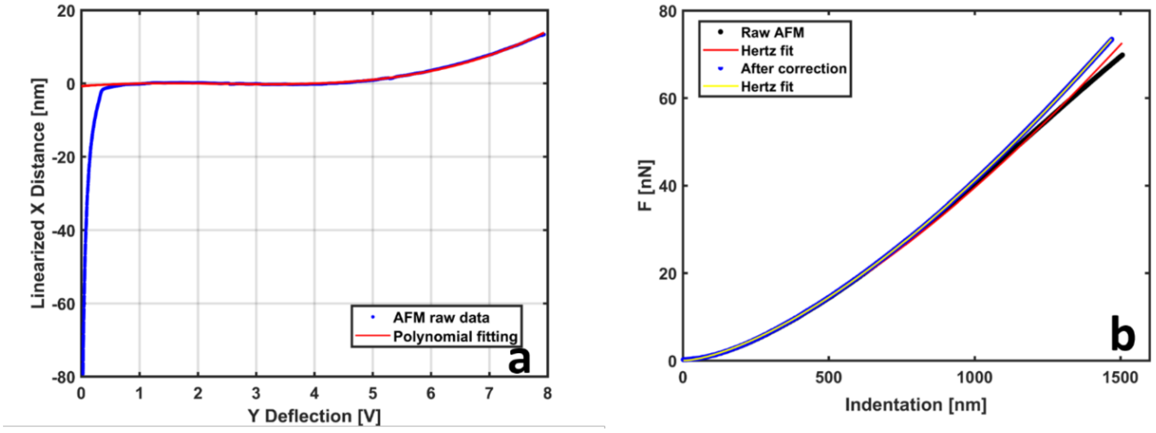

Supplementary Figure 3 | Non-linearity correction for photo-detector. (**a**) Example of force curve on rigid substrate after linearization, showing non-linearity between 5V and 8V deflection voltage signal. A 4th order polynomial fitting is used for correction. (**b**) Comparison between raw and corrected AFM nanoindentation data on 30 kPa hydrogel using Hertz fitting procedure.

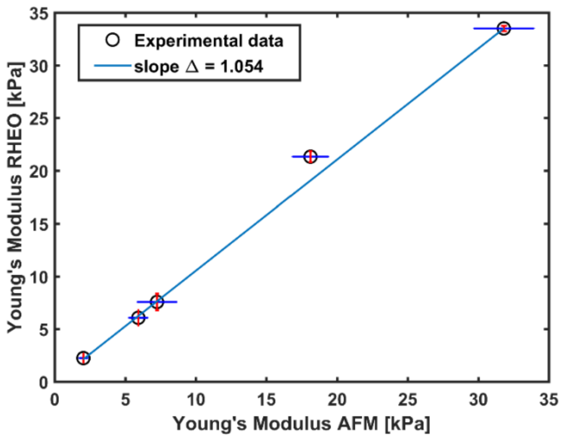

Supplementary Figure 4 | Application of SNAP procedure. Example of linear regression between Young's moduli by colloidal probe AFM and rheology. The slope represents the correction parameters to be applied in SNAP for the optical lever sensitivity. Errors bars in rheological data represent the standard deviations obtained on 4 different replicates, while errors for AFM represent the quadrature sum of single measurements uncertainty and standard deviation on 10 replicates obtained at different macroscopic positions.

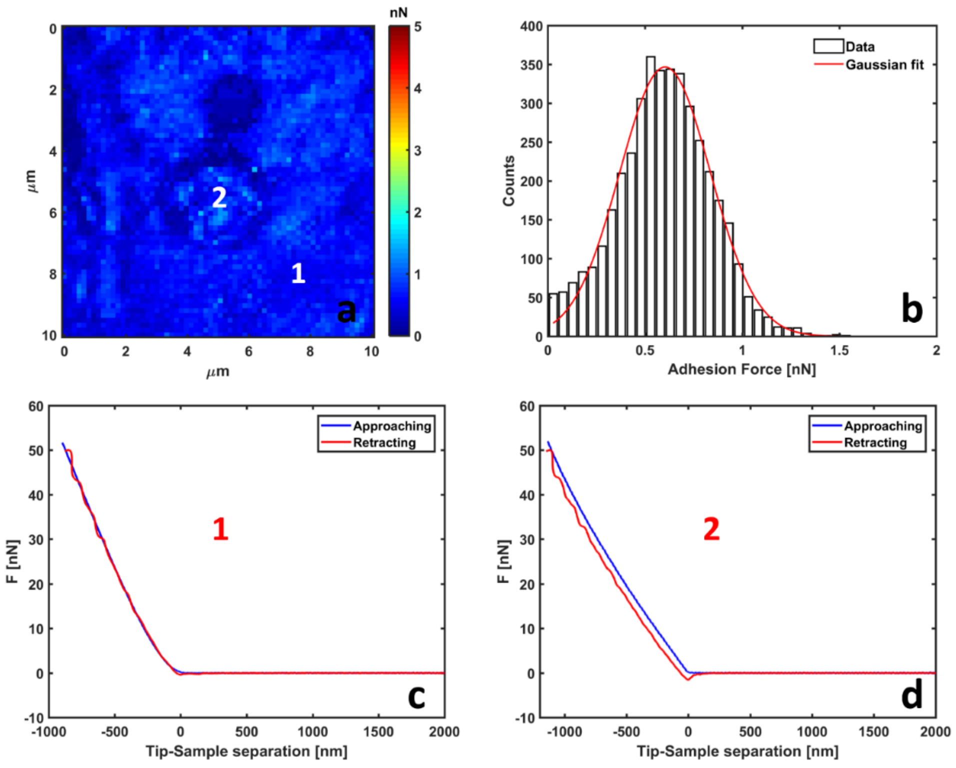

Supplementary Figure 5 | Probe-Sample adhesion analysis. (**a**) Adhesion force map of 1µ-gel produced during analysis of Figure 3 of main text. (**b**) Quantitative analysis using histogram and Gaussian fitting. Complete force curves from map (**a**), approaching and retracting parts, are presented for point 1 (**c**) and point 2 (**d**).

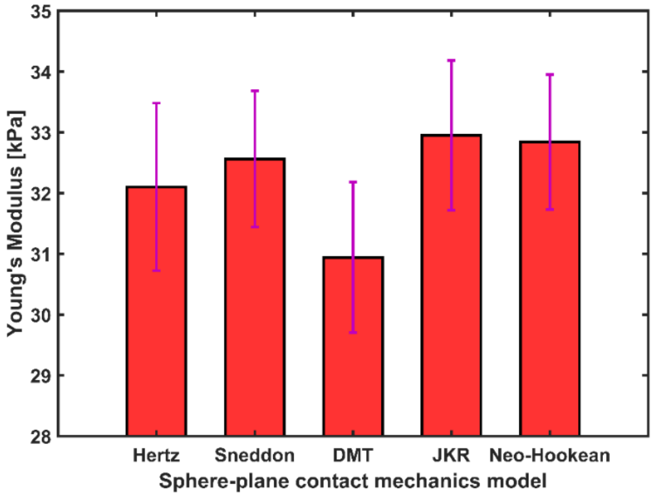

Supplementary Figure 6 | Contact mechanics model comparison. Young's modulus calculated using different contact mechanics models considering approximate Hertz, Sneddon's exact solution, adhesion (DMT and JKR) and non-linearity. Error bars represent the standard deviation calculated by Gaussian fitting on histogram for a single AFM force volume.

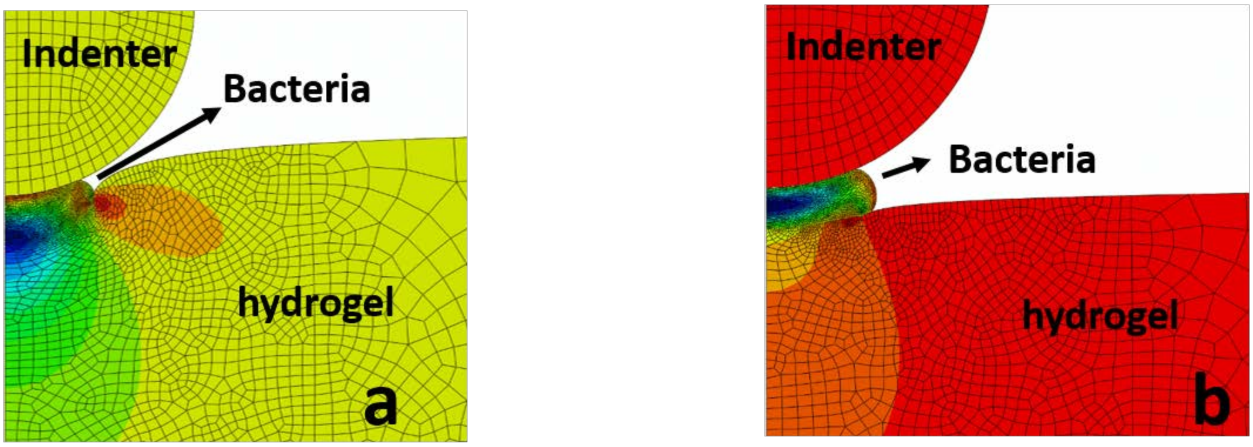

Supplementary Figure 7 | Finite Element Simulation results on bacteria supported on gels. Stress-Deformation fields along vertical direction for model on (a) E. Coli 6 kPa gel, (b) E. Coli on 30 kPa gel.

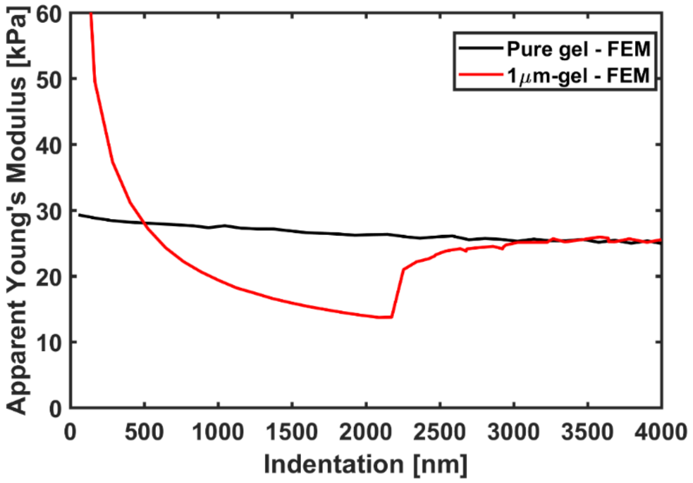

Supplementary Figure 8 | Apparent Young's by finite element simulation on pure gel and 1μm-gel. Pure simulation results using an extended indentation axis to show clearly the 3 trends of apparent modulus described in main text. Same simulations are presented in Figure 3 (main text) on a shorter x-axis (0 - 1200 nm) to follow AFM data.

Supplementary Tables

	Pure gel	2.5μ-gel	1μ-gel	0.15μ-gel
NIPAM (g)	1.200	1.200	1.200	1.200
BIS (mg)	60	60	60	60
Water (ml)	15	15	15	15
TEMED (mg)	46.63	46.63	46.63	46.63
APS (mg)	35.94	35.94	35.94	35.94
PS μ–spheres (mg)	0	60[a]	60[b]	60[c]
Conversion (%)	100	100	100	100
Appearance	transparent	opaque	opaque	opaque

Supplementary Table 1 | Synthesis of composite poly(N-isopropylacrylamide) gels. Hydrogels in presence of same concentration of polystyrene microspheres having different radii, R = 2.5 μm (a), 1 μm (b), 0.15 μm (c). Polymerization temperature = 5°C, polymerization time = 12h. Conversion (%) was determined gravimetrically after drying under vacuum at 50 °C for 72h after dialysis.

Supplementary Methods

Supplementary Method 1 | Scanning electron microscopy. After gold sputtering, SEM images were recorded using a HITACHI SU-70 instrument at an acceleration voltage of 5 kV under vacuum. Supplementary Figure 1 (a): R = 0.15 μm, (c): R = 1 μm, e: R = 2.5 μm) shows the SEM surface morphology of monodisperse polystyrene spheres on glass before embedding. After synthesis, the composite gels were first swollen in deionized water at 20 °C for 24 h to equilibrate and then freeze dried under vacuum. Supplementary Fig. 1 (b: 0.15μ-composite, d: 1μ-composite, f: 2.5μ-composite) shows the surface of gel focused on regions with single spheres protruding from the flat surface. The composite structure shows random dispersion with negligible aggregation of spheres (due to the low volume filler concentration) and continuity at the interface between spheres and gel matrix.

Supplementary Method 2 | Rheology. The rheological experiments were performed using an Anton Paar MCR 302 rheometer, equipped with a Peltier heater and a solvent trap. In order to avoid slippage, the samples were superglued to the surface of the lower plate and the 8 mm upper plate. To avoid problems inaccuracy from having too low gaps due to superglue layers, gaps of 3-4 mm were used, depending on the thickness of the water-swollen sample. The loaded sample was chosen to be significantly larger than the 8 mm geometry. After the superglue had dried (ca. 1 min), it was cut in shape by a razorblade – thus avoiding significant artefacts due to superglue covering the outer sample surface. All experiments were performed, while immersed in deionized water, thus preventing artefacts due to drying out of the sample. Temperature ramps were performed with a profile of starting at T=1°C, going to 50°C and then returning back to 1°C at a heating/cooling rate of 1 $Kmin^{-1}$, while measuring at an angular frequency of ω=10 s^{-1} at a deformation γ_0=1 %. Due to the nature of hydrogels being incompressible solids[1] and the validity of the Cox-Merz rule,[2] it was possible to convert shear into elongational modulus by $3|G^*|=E$,[3] thus, allowing for comparing shear modulus and AFM-modulus. Strictly spoken, one needs to be careful about the application of the Trouton rule, [3] as it strictly spoken is only valid for unfilled materials. However, for small deformations and low filler contents, as used here, it is nevertheless valid as well.

Supplementary Notes

Supplementary Note 1 | Materials preparation. *N*-isopropylacrylamide (NIPAM, Aladdin) was recrystallized from n-hexane. *N*,*N*-methylene bisacrylamide (BIS, Macklin), *N*,*N*,*N'*,*N'*-tetramethyl ethylenediamine (TEMED, Aladdin), Ammonium persulfate (APS, Aladdin) were used as received. Distilled water was obtained by re-distillation of distilled water. Commercially available monodisperse microspheres of polystyrene (PS-μ–spheres) were purchased from EPRUI Biotech Co. Ltd in 3 different radii: 2.5 μm, 1 μm, 0.15 μm.

A solution of TEMED in water with concentration of 107 $mmolL^{-1}$ and a solution of APS in water having concentration of 42 $mmolL^{-1}$ were prepared. At first, NIPAM (1.2 g), BIS (60 mg) and TEMED (3.75 ml) solution and solvents were taken in 25 mL glass tube (25 mm internal diameter × 100 mm length) fitted with a rubber septum. The pre-gel mixtures (NIPAM, BIS, and TEMED) in water were purged with N_2 gas for 30 min. These mixtures were dipped into an isothermal bath and maintained at 5 ± 0.5 °C under N_2 atmosphere to equilibrate. Different heterogeneous gels were prepared adding to the mixture same weight (60 mg, 5% of monomer weight) of PS-μ–spheres with different sizes. Then, the APS stock solution (1.875 ml) was purged with N_2 gas at 5 °C for 30 min. After that, the N_2 purged APS stock solution was added to the pre-gel mixture through a rubber septum by a degassed syringe, mixed immediately and allowed to react at 5 °C for 12 hrs. The prepared gels were cut into small square type shapes (approximately 6x6 mm^2 and 3 mm thickness), and dipped into the de-ionized water for dialysis to remove the unreacted chemicals, and water were changed twice a day for 7 days. After the dialysis, the gels were dried under vacuum at 50 °C for 72 hours. Nearly 100% conversion was determined gravimetrically for all prepared samples.

Chitosan (CS) with a viscosity-average molecular weight of 5.0×10^4 was purchased from Zhejiang Golden-Shell Pharmaceutical Co. Ltd. (China). N,N'-Methylenebisacrylamide (MBA, 99.5%)， and hydroxyethyl acrylate) (HEA,99%) were purchased from Energy Chemical (Shanghai, China).

Chitosan powder (0.5 g) was completely dissolved in acetic acid/water solution (2 %, 16 ml) under the continuous stirring for 24 hours, and then the mixture was cooled in an ice-water bath. 2 mL glutaraldehyde/water solution (2 wt %) was added under fast stirring for 30 seconds, then the cross-linked CS mixture was transferred to prepared quartz mold and kept at room temperature for 24h, the CS-gel was obtained. The first CS network was prepared according to preparation process of CS hydrogel with four different contents (1ml, 2ml, 3ml, 4ml) of glutaraldehyde/water solution (2 wt %). And the first CS network as named as CSx-y, here x was the concentration of glucosamine structural unit, and y was the percentage of NH_2 group that participated in the crosslinking reaction. After then, the CS first network was dipped in the 100.0 ml mixture solution containing HEA/water solution (2 mol L^{-1}), MBA (0.1 mmol) and α-ketoglutaric acid (0.2 mmol) for 24 h to reach the equilibrium swelling state. Then the hydrogel was transferred to prepared

quartz mold, then CS-PHEA double-network hydrogels (CS-PHEA gel, abbreviated as CPx-y gel) with different crosslinking degree were obtained after irradiated under ultraviolet light of 365 nm (300W) for 8h at 200 mW cm^{-2}.

The hydrogels were cut to pieces with 20 mm × 20 mm, and they were sterilized with ethanol (70%), afterwards, 0.2 ml solution of bacteria (2×108 CFU ml^{-1}) was added to the hydrogels surface and covered by a polyethylene (PE) film (30 mm×30 mm). The bacteria were then incubated for 24 h at 37±1 °C and a relative humidity (RH) of 90%. Two samples were used as bacteria substrates, called CP3-0.8 and CP3-0.2 respectively hard and soft hydrogel substrates.

Supplementary Note 2 | Achieving high precision with AFM calibration and corrections. Radius and status of spherical colloidal probes (Supplementary Fig. 2b) were measured and controlled by reverse imaging on TGT1 calibration grating (NT-MDT) made up of close packed pyramids with a tip angle 50° and a height 0.5 μm, shown in Supplementary Fig. 2a. Imaging was performed in contact mode at 4096x1024 resolution, collecting 3-4 images for statistics purpose. The convolution effect between probe and spikes is leading to repetition of probe apex morphology for each spike (reverse imaging) as shown in Supplementary Fig. 2c. Reverse imaging after measurements on hydrogels shows also absence of contamination at the nanoscale. Statistical data analysis of several spherical caps (Supplementary Fig. 2d) leads to the tip radius calibration R, fitting the data collection with the relation between volume V and height h as shown in the following equation: [4]

$V = \frac{\pi}{3} h^2 (3R - h)$ Equation 1

The procedure used the radius calibration method was fully documented by Indrieri et al.[4]

All AFM operations involve detection of cantilever deflection through laser beam reflected on a segmented photodetector. Photodetector response around the center (zero voltage) is linear but laser spot near edges show non-linearity towards saturation at 12V (maximum photodetector voltage), therefore, restricting the usable voltage range on photodetector. Supplementary Fig. 3a shows typical force curve on rigid substrate with non-linear response for deflection signals from 5 to 8 V. The correction is performed by fitting the non-linear part using a 4th degree polynomial law and subtracting it from raw data. Correction is generated on force curves on rigid substrate where indentation is negligible. Finally, the correction is transported on experimental data with indentation where, generally, non-linearity causes a forward bending and underestimation of Young's moduli. Examples of correction on indentation curves on 30 kPa hydrogels are presented in Supplementary Fig. 3b showing improved performance of Hertz fit after correction all over the indentation range.

Standardized Nanomechanical procedure (SNAP) was recently introduced,[5] discussing problematics in nanomechanical measurements experienced in 11 different AFM labs across Europe and participating to COST Action TD1002, European network on applications of atomic force microscopy to nanomedicine and life sciences. The main issue was about the error/imprecision during optical lever sensitivity calibration, then propagating in thermal noise calculation. Through an external, independent measurement of elastic constant of cantilever, z_{sens} can be corrected using the following relations:

$$z_{sens}^{new} = \lambda * z_{sens}^{old} \qquad \lambda = \sqrt{\frac{k_{old}}{k_{external}}} \qquad (2)$$

As external and independent measurements in place of vibrometer or interferometer we use 5 calibrated samples with rheometer. After the confirmation of quantitative characterization with colloidal probes,[6] we decided to prepare and array of 5 homogeneous gels with moduli equally distributed between 1 and 30 kPa in order to perform the calibration of the probe. So far, recent works from our group demonstrated how the series of well-defined and homogenous gels show Young's modulus at micro-nanoscale in excellent agreement with rheological macroscopic measurements, highlighting their importance for calibration purpose.[6-8]

Supplementary Fig. 4 shows the comparison between Young's moduli measured by rheology and colloidal probe AFM. Due to the linear relationship between moduli and applied force, the slope coefficient can be used to correct elastic constant and optical lever sensitivity using Eq.2, as reported in SNAP.[5] The method is independent of intrinsic AFM optical alignment, still based on rheometer accuracy.

Supplementary Note 3 | Adhesion Analysis. Regarding the samples under investigation (hydrogels and bacteria), especially working completely immersed in water solution, a negligible adhesion was detected. 'AFMech Suite' software allows focusing on adhesion, specifically, on single force spectroscopy data showing that adhesion is detectable but negligible in comparison with the indentation forces. This is a crucial point for the validity of Hertz model used for data analysis. Adhesion analysis was performed for all sample investigated, here, we present the analysis in correspondence to Figure 3 of main text. Adhesion map is shown in Supplementary Fig. 5a confirming low adhesion all over the surface sample. Quantitative analysis in Supplementary Fig. 5b shows an average adhesion force of F_{AD} = 0.6 ± 0.2 nN, around 1% of maximum indentation force, therefore, ensuring validity of Hertz model.

Examples of complete force curves (approaching and retracting) are shown Supplementary Fig. c for bulk gel and Supplementary Fig. d on top of spherical inclusion. The contact point for the retracting curve is calculated exactly like approaching curve for a better comparison. An inset in each graph shows a magnification on the

adhesion between tip and samples. In the case presented in Supplementary Fig. d, for 1μ-gel, the adhesion well reaches 1.2 nN of intensity. The higher adhesion on exposed spherical inclusion is indicative of residual surface polymeric chains hanging at the surface of inclusion after cutting procedure. A spherical probe with big contact area is enhancing the probability to catch such a semi-cross-linked chain.

Supplementary Note 4 |Spherical Indentation Models. As discussed in Methods, Hertz model for a sphere indenting a plane is the simplest contact mechanics model, affected by several constraints: sample must be linear elastic, homogeneous, isotropic, showing negligible viscosity and adhesion. Thickness of sample must be large compared with indentation $h >> \delta$, while same indentation must be small in comparison with probe radius $\delta << R$.

The main scope of the work is evaluating the transition from homogeneous to heterogeneous samples, still a justification and validation in using Hertz model is necessary.

1. Hydrogel samples are isotropic. Changing direction during cutting did not influence moduli values either in rheology and AFM
2. Non-linear elasticity behavior is negligible for shallow indentation. Hydrogels and bacteria are generally treated as hyperleastic (stress and strain non-linear) following Neo-Hookean model.[9] In this framework, under spherical indentation, the relations between the force based on Neo-Hookean model and Hertz solution was provided by Zhang et al.[10] and confirmed in our recent work.[11] In current work, R = 2500 nm, and the maximum indentation during AFM experiments is around 800 nm, which leads to an error of 3% accordingly.
3. Hydrogels used here show purely elastic behavior and negligible viscosity. This is directly confirmed by rheology measurements. Moreover AFM measurements did not show frequency dependence (see Galluzzi et al.[6]) and there is no relaxation hysteresis between approaching and retracting indentation curves (see Supplementary Fig. 5).
4. Adhesion of hydrogels samples in solution is negligible as shown in previous section.
5. Sample thickness is macroscopic (in millimeter range), while radius and indentation characteristic scale are in micrometer range. Even if in our study is not necessary, 'AFMech Suite' grants the possibility to apply Finite Thickness correction (see 'AFMech Suite' guide).
6. Indentation is small compared with probe radius. Sneddon spherical model is describing the exact relation of a sphere indenting pure elastic plane, still Hertz is a good approximation at shallow indentations. Following the work of Puricelli et al.[12] , for spheres with R = 2.5 µm in cases of E = 30 kPa and F = 40 nN, the relative discrepancy between the two models is well below 3%.

'AFMech Suite' allows choosing several geometries and models. Here, focusing on sphere geometry, Hertz, Sneddon (sphere) and adhesive DMT, JKR and non-linear hyperelastic Neo-Hookean models are compared in Supplementary Fig. 6 directly analyzing experimental data of FV measurement on homogeneous hydrogel (30 kPa).

Equations relative to this study are reported as follows:

Hertz $$F = \frac{4}{3}\frac{E\sqrt{R}}{(1-\nu^2)}\delta^{3/2}$$ (3)[13]

Sneddon $$F = \frac{E}{2(1-\nu^2)}\left[(a^2 + R^2)\ln\left(\frac{R+a}{R-a}\right) - 2aR\right] \qquad \delta = \frac{1}{2}a\ln\left(\frac{R+a}{R-a}\right)$$ (4)[14]

DMT $$F = \frac{4}{3}\frac{E\sqrt{R}}{(1-\nu^2)}\delta^{3/2} + F_{\mathrm{AD}}$$ (5)[15]

JKR $$F = \frac{4Ea^3}{3R(1-\nu^2)} - \sqrt{\frac{16EF_{\mathrm{AD}}}{3(1-\nu^2)}a^3} \qquad \delta = \frac{a^2}{R} - \sqrt{\frac{4F_{\mathrm{AD}}(1-\nu^2)}{ER}a}$$ (6)[16]

Neo-Hookean $$F = \frac{4}{3}\frac{E\sqrt{R}}{(1-\nu^2)}\delta^{3/2}\left(1 - 0.15\frac{\delta}{R}\right)$$ (7)[10]

Where *F*, *E*, *R*, ν, δ, *a* and F_{AD} represent respectively force, Young's modulus, radius of spherical probe, Poisson's ratio, indentation, radius of contact area and adhesion force.

Besides DMT model, usually employed for systems showing strong capillary adhesion (our system is completely immersed in solution, thus making one of the key assumptions of this model invalid), all models show comparable Young's moduli values, well inside experimental error. Hertz approximation validity was confirmed directly, allowing to focus our discussion in solving heterogeneity problems.

Supplementary References

1 Ferry, J. D. *Viscoelastic Properties of Polymers*. (John Wiley and Sons, 1980).

2 Cox, W. P. & Merz, E. H. Correlation of dynamic and steady flow viscosities. *J. Polym. Sci.* **28**, 619-622, doi:10.1002/pol.1958.1202811812 (1958).

3 Trouton, F. T. On the viscous traction and its relation to that of viscosity. *Proc. Roy. Soc.* **77**, 426 (1906).

4 Indrieri, M., Podestà, A., Bongiorno, G., Marchesi, D. & Milani, P. Adhesive-free colloidal probes for nanoscale force measurements: Production and characterization. *Rev. Sci. Instrum.* **82**, 023708, doi:10.1063/1.3553499 (2011).

5 Schillers, H. *et al.* Standardized Nanomechanical Atomic Force Microscopy Procedure (SNAP) for Measuring Soft and Biological Samples. *Sci. Rep.* **7**, 5117, doi:10.1038/s41598-017-05383-0 (2017).

6 Galluzzi, M. *et al.* Space-resolved quantitative mechanical measurements of soft and supersoft materials by atomic force microscopy. *NPG Asia Mater.* **8**, e327, doi:10.1038/am.2016.170 (2016).

7 Wang, Q. *et al.* Random copolymer gels of N-isopropylacrylamide and N-ethylacrylamide: effect of synthesis solvent compositions on their properties. *RSC Adv.* **7**, 9381-9392, doi:10.1039/C6RA27348C (2017).

8 Biswas, C. S. *et al.* Versatile Mechanical and Thermoresponsive Properties of Macroporous Copolymer Gels. *Macromol. Chem. Phys.*, 1600554, doi:10.1002/macp.201600554 (2017).

9 Stadler, F. J., Friedrich, T., Kraus, K., Tieke, B. & Bailly, C. Elongational rheology of NIPAM-based hydrogels. *Rheol. Acta* **52**, 413-423, doi:10.1007/s00397-013-0690-x (2013).

10 Zhang, M. G., Cao, Y. P., Li, G. Y. & Feng, X. Q. Spherical indentation method for determining the constitutive parameters of hyperelastic soft materials. *Biomech. Model. Mechanobiol.* **13**, 1-11, doi:10.1007/s10237-013-0481-4 (2014).

11 Tang, G., Galluzzi, M., Biswas, C. S. & Stadler, F. J. Investigation of micromechanical properties of hard sphere filled composite hydrogels by atomic force microscopy and finite element simulations. *J Mech. Behav. Biomed. Mater.* **78**, 496-504, doi:10.1016/j.jmbbm.2017.10.035 (2018).

12 Puricelli, L., Galluzzi, M., Schulte, C., Podesta, A. & Milani, P. Nanomechanical and topographical imaging of living cells by atomic force microscopy with colloidal probes. *Rev. Sci. Instrum.* **86**, 033705, doi:10.1063/1.4915896 (2015).

13 Borodich, F. M. The Hertz-Type and Adhesive Contact Problems for Depth-Sensing Indentation. **47**, 225-366, doi:10.1016/b978-0-12-800130-1.00003-5 (2014).

14 Sneddon, I. N. The relation between load and penetration in the axisymmetric boussinesq problem for a punch of arbitrary profile. *Inter. J. Eng. Sci.* **3**, 47-57, doi:10.1016/0020-7225(65)90019-4 (1965).

15 Derjaguin, B. V., Muller, V. M. & Toporov, Y. P. Effect of contact deformations on the adhesion of particles. *J. Colloid Interf. Sci.* **53**, 314-326, doi:10.1016/0021-9797(75)90018-1 (1975).

16 Johnson, K. L., Kendall, K. & Roberts, A. D. Surface Energy and the Contact of Elastic Solids. *Proc. Roy. Soc. A* **324**, 301-313, doi:10.1098/rspa.1971.0141 (1971).
