# Supplementary material for: Atomic force microscopy methodology and AFMech Suite software for nanomechanics on heterogeneous soft materials
Source: Nat Commun. 2018 Sep 4;9:3584. doi: 10.1038/s41467-018-05902-1 (PMC6123404; doi:10.1038/s41467-018-05902-1)
Supplement: Supplementary file 3 — Description of Additional Supplementary Files [file 41467_2018_5902_MOESM3_ESM.pdf]

Description of Additional Supplementary Files

File Name: Supplementary Software 1
Description: AFMech Suite is a software composed by 5 interacting interfaces in order to analyse AFM indentation data connected to morphology such as Force Volume or Force Mapping. This suite is written in Matlab language using event/object programming to provide an alternative tool for basic or advanced analysis of AFM force volume. The analysis is real-time allowing the user to be in control in each step of analysis and eventually interact (changing parameters, graphical representations) during the process. The standard user should be from AFM beginner level to expert level with AFM nanomechanics experience, anyway the graphical user interface does not require knowledge of Matlab language.
